# Are TaNAC Transcription Factors Involved in Promoting Wheat Yield by *cis*-Regulation of *TaCKX* Gene Family?

**DOI:** 10.3390/ijms25042027

**Published:** 2024-02-07

**Authors:** Adnan Iqbal, Joanna Bocian, Mateusz Przyborowski, Wacław Orczyk, Anna Nadolska-Orczyk

**Affiliations:** Plant Breeding and Acclimatization Institute—National Research Institute, Radzikow, 05-870 Blonie, Poland

**Keywords:** *TaNACs*, *TaCKX* GFMs, bread wheat, yield-related traits, *cis*-regulation, transcription factors

## Abstract

NAC transcription factors (TFs) are one of the largest TF families in plants, and TaNACs have been known to participate in the regulation of the transcription of many yield-regulating genes in bread wheat. The *TaCKX* gene family members (GFMs) have already been shown to regulate yield-related traits, including grain mass and number, leaf senescence, and root growth. The genes encode cytokinin (CK) degrading enzymes (CKXs) and are specifically expressed in different parts of developing wheat plants. The aim of the study was to identify and characterize *TaNACs* involved in the *cis*-regulation of *TaCKX* GFMs. After analysis of the initial transcription factor data in 1.5 Kb *cis*-regulatory sequences of a total of 35 homologues of *TaCKX* GFMs, we selected five of them, namely *TaCKX1-3A*, *TaCKX22.1-3B*, *TaCKX5-3D*, *TaCKX9-1B*, and *TaCKX10*, and identified five *TaNAC* genes: *TaNACJ-1*, *TaNAC13a*, *TaNAC94*, *TaNACBr-1*, and *TaNAC6D*, which are potentially involved in the *cis*-regulation of selected *TaCKX* genes, respectively. Protein feature analysis revealed that all of the selected TaNACs have a conserved NAC domain and showed a stable tertiary structure model. The expression profile of the selected *TaNACs* was studied in 5 day-old seedling roots, 5–6 cm inflorescences, 0, 4, 7, and 14 days-after-pollination (DAP) spikes, and the accompanying flag leaves. The expression pattern showed that all of the selected *TaNACs* were preferentially expressed in seedling roots, 7 and 14 DAP spikes, and flag leaves compared to 5–6 cm inflorescence and 0 and 4 DAP spikes and flag leaves in Kontesa and Ostka spring wheat cultivars (cvs.). In conclusion, the results of this study highlight the potential role of the selected *TaNACs* in the regulation of grain productivity, leaf senescence, root growth, and response to various stresses.

## 1. Introduction

Bread wheat (*Triticum aestivum* L.) is the third most important cereal crop grown around the world after maize and rice. It has a complex allohexaploid (AABBDD) genome, which contains three: A, B, and D diploid subgenomes [1,2,3]. The complexity of the bread wheat genome is challenging in molecular and genetic research and breeding; however, on the other hand, it is a large reservoir of homoeologous genes, which can be targeted for breeding [4]. As the world’s population grows, the demand for high yielding wheat cultivars (cvs.) is increasing [5]. However, climate change, biotic, and abiotic stresses pose a parallel challenge to the productivity of wheat crop [6,7]. Therefore, functional characterization of yield-related genes could be a way to address this challenge.

Cytokinins (CKs) belong to the group of plant hormones that have direct implications on plant growth and development [8,9]. They have also been known to respond to biotic and abiotic stresses, including maintaining mineral nutrition and leaf senescence [10,11,12]. The primary role of cytokinins is to promote cell division and that of auxins is to promote cell expansion, while the balance between the concentrations of cytokinins and auxins controls the growth of the root and shoot [13] A reduced concentration of cytokinins promotes tillering, while inhibited by auxins [14,15]. In a recent review cytokinin was indicated as a key factor of seed yield [8]. The phytohormone is produced locally in the roots and aerial parts of the plants or is transported from the roots to the shoots [16,17]. The cytokinin content in the wheat spikes directly contributes to grain yield and thousand-grain weight (TGW), as well as the chlorophyll content in flag leaves and the seedling root weight [15,18,19,20,21].

*CKX* gene family members (GFMs) encode an enzyme named oxidase/dehydrogenase that irreversibly degrades cytokinin and hence regulates the content and functions of cytokinin in various parts of the plant [14]. According to the most recent numbering, there are 13 basic numbers of *TaCKX* GFMs in allohexaploid bread wheat. Eleven of them have homoeologous genes in the three subgenomes (A, B, D) and two are located exclusively in subgenome D. Therefore, the final number of all homoeologous genes is 35 [14]. The results of the study conducted by Ogonowska et al. (2019) [22] showed that the expression of *TaCKX* GFMs is developmentally and organ-specific. For example, considering the new numbering of *TaCKX* GFMs, the expression of *TaCKX7*, *8*, and *10* (previously *9*) was highly specific to seedling roots; expression of *TaCKX4*, *5*, and *9* (previously *11*) was specific to leaves. However, expression of *TaCKX1*, *TaCKX2* genes was specific to inflorescences and spikes, and finally *TaCKX3* (previously *6*), *8* (previously *11*), and *11* (previously *3*), were expressed at various levels in all of the organs tested. The results of several studies have shown that *TaCKX* GFMs influence the tiller number, weight of seedling roots, grain size, thousand-grain weight (TGW), spike phenotype, and phytohormonal homeostasis in bread wheat [18,19,20,21,23,24]. Expression of a single *TaCKX* is co-regulated by the other *TaCKX* GFMs or genes encoding TFs; for example, RNA interference-based silencing (RNAi) of *TaCKX1* downregulated the expression of *TaCKX2* genes and upregulated the expression of *TaCKX5* and *TaNAC2-5A* [18]. Similarly, RNAi silencing of *TaCKX2* coregulated the expression of *TaCKX1* antagonistically by downregulation of *TaCKX1*, but upregulation of *TaCKX5* and *TaNAC2-5A* [18]. Similarly, strong silencing of *TaCKX2.2.2* slightly reduced the expression of *TaCKX2.2.1* and greatly decreased the expression of *TaCKX5*, and *TaCKX11*; however, the expression of *TaCKX1*, *TaCKX2.1*, and *TaCKX9* remained unchanged [20]. The coregulation of *TaCKX* GFMs and the expression pattern also influenced phytohormonal homeostasis and yield-related traits in wheat crops, such as grain size, thousand-grain weight (TGW), and chlorophyll content in flag leaves [18,19,20]. The role of *TaCKX* GFMs in yield-related traits is well documented; however, the transcription regulation of this gene family by transcription factors is still largely unknown. Recently, it has been reported that *TaCKX* GFMs have been found to coregulate the expression of gene encoding transcription factor containing the NAC domain [25]. The RNAi silencing of *TaCKX2* genes upregulated the expression of a *TaNAC2-5A* [18].

Transcription factors play an important role in gene networking to control plant growth, development, and adaptation to the environment [26]. NAC TFs (NAM, ATAF, and CUC) are among the largest TF families which are known to regulate genes by interacting with DNA binding sites in promoter sequences or upstream sequences to the promoter sequences, called *cis*-regulatory sequences. There are 263 TaNAC TFs in bread wheat and these TFs have been known to control biotic and abiotic stresses along with senescence, morphogenesis, and phytohormonal homeostasis by *cis*-regulation of various genes, as reviewed by Iqbal et al. (2022) [25].

Seed storage proteins (SSPs) are important for the elasticity of wheat flour. TaNAC77 TF binds to the promoter sites of the *SSP* encoding genes and regulates gene transcription. Additionally, silencing of the *TaNAC77* reduced 24% of the expression of *SSP* encoding gene [23]. Similarly, TaSPR is a novel NAC domain transcription factor from common wheat (*Triticum aestivum*). Overexpression of *TuSPR* reduced the total content of SSPs up to 15.97% in *Triticum urartu* and knock-down of *TaSPR* increased the SSP content up to 20.34% [27]. TaNAC100 also interacts with the *cis*-element of *TaGBSS1* and *TaSUS2* and regulates the synthesis of seed proteins and starch. Overexpression of *TaNAC100* significantly reduced the total seed proteins including SSPs [28]. Leaf senescence is another important yield and crop productivity-related trait, which affects the gain-filling trait of the crop. The NAC domain containing *NAM-1* alleles is involved in delayed leaf senescence that causes an extended grain filling time [29]. Rice OsNAC2 TF modulates root development by *cis*-regulation of two auxin-regulating genes (*GH3.6* and *GH3.8*) and one cytokinin-related gene (*OsCKX4*), and knock-down of *OsNAC2* significantly improved root hairs and length [30]. TaNAC2-5A has been reported to be a nitrate-inducible transcription factor, and overexpression of *TaNAC2-5A* positively influenced root growth, higher concentration of nitrogen in grains, and overall grain yield [31]. Furthermore, TaNAC2-5A has been reported to interact with the *TaNRT2.5-3B* promoter region and help establish seed vigor [32].

Taking into account the significant role of *TaCKX* GFMs in controlling yield-related traits in wheat and the possible role of *TaNAC* genes in the regulation of these genes, we performed research on the identification and characterization of these *TaNACs* that interact with selected *TaCKX* GFMs. The research was conducted on two wheat cultivars (cvs.), Kontesa and Ostka. The results of this study suggest that selected *TaNACs* could be candidate genes for the regulation of plant growth and development, yield-related traits, and stress management.

## 2. Results

### 2.1. Identification of TaNAC Transcription Factor Binding Sites in TaCKX GFMs cis-Regulatory Sequences

We retrieved 1.5 kb upstream *cis*-regulatory sequences of 35 *TaCKX* GFMs, listed in Table S3, from the wheat ensemble database (wheat EnsemblPlants v2.1). The sequences were analyzed to identify TF binding sites from the PlantTFDB v5.0 plant transcription factor database.

After running TF data, the results of all transcription factor binding sites on our given *cis*-regulatory sequences of *TaCKX* GFMs were downloaded and compiled as a raw FIMO file (Folder S1). From the raw FIMO files of the plant transcription factors, we identified and sorted TaNAC transcription factors and their *cis*-regulatory binding sites on *TaCKX* GFM sequences. The results were summarized in an Excel sheet and, due to space constraints, the results were provided as File S1. Based on the highest score, the lowest p′ and q′ values of TaNAC transcription factors, and our previous experience with *TaCKX* GFMs for their role in improving wheat yield [18,19,20,21,22,24], we selected five *TaCKX* GFMs; these being, *TaCKX1-3A*, *TaCKX2.2.1-3B*, *TaCKX5-3D*, *TaCKX9-1B*, and *TaCKX10-7B*.

### 2.2. Sequencing of Selected TaCKX GFM cis-Regulatory Regions from Kontesa and Ostka Cultivars

**Figure 1:**
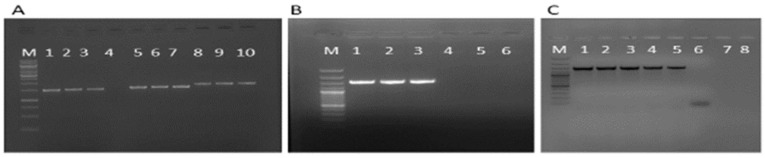
PCR amplification analysis of the selected *TaCKX* GFM cis-regulatory sequences. (**A**) M: 1 kb plus marker; Lanes 1 and 2: *TaCKX1-3A* Kontesa and Ostka, respectively; Lane 3: positive control from previously optimized PCR; Lane 4: negative control (H_2_O); Lanes 5 and 6: *TaCKX5-3D* Kontesa and Ostka, respectively; Lane 7: positive control from previously optimized PCR; Lanes 8 and 9: *TaCKX 2.2.1-3B* Kontesa and Ostka, respectively; Lane 10: positive control from previously optimized PCR. (**B**) M: 100 bp plus marker; Lanes 1 and 2 *TaCKX10-7B* Kontesa and Ostka, respectively; Lane 3: positive control from previously optimized PCR; Lane 4: negative control (H_2_O); Lanes 5 and 6: empty. (**C**) M: 100 bp plus marker; Lanes 1–2 and 3–4 *TaCKX9-1B* Kontesa and Ostka, respectively; Lane 5: positive control from previously optimized PCR; Lane 6: negative control (H_2_O); Lanes 7 and 8: empty.

The PCR fragments of five selected *TaCKX* GFMs, i.e., *TaCKX1-3A*, *TaCKX22.1-3B*, *TaCKX5-3D*, *TaCKX9-1B*, and *TaCKX10-7B*, were amplified as 1500 bp, 1561 bp, 1549 bp, 1401 bp, and 1538 bp, respectively, from the total genomic DNA of the Kontesa and Ostka cultivars (cvs.) (Figure 1A–C). The amplicons were cloned into easy pGEMT vectors and sequenced. The sequences were deposited in the NCBI database under the accession numbers PP078647, PP078649, PP078651, PP078653, PP078655 and PP078648, PP078650, PP078652, PP078654, and PP078656 belonging to the *TaCKX1-3A*, *TaCKX22.1-3B*, *TaCKX5-3D*, *TaCKX9-1B*, and *TaCKX10-7B* of the Kontesa and Ostka cvs., respectively. These sequences were further analyzed for conserved motif analysis and TaNAC TF identification.

### 2.3. Mapping of Conserved Motifs in the Kontesa and Ostka Cultivars

**Figure 2:**
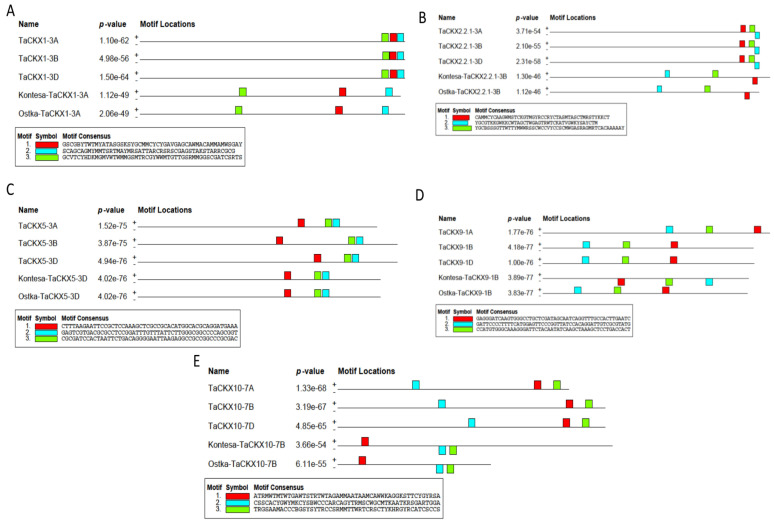
Comparison of the conservative motifs of the *TaCKX1*-*3A* (**A**), *TaCKX22.1*-*3B* (**B**), *TaCKX5*-*3D* (**C**), *TaCKX9*-*1B* (**D**), and *TaCKX10*-*7B* (**E**) GFMs from the Kontesa and Ostka cultivars to that identified in the wheat reference genome. (A = Adenine, C = Cytosine, G = Guanine, T = Thymine, U = Uracil, R = Guanine/Adenine (purine), Y = Cytosine/Thymine (pyrimidine), K = Guanine/Thymine, M = Adenine/Cytosine, S = Guanine/Cytosine, W = Adenine/Thymine, B = Guanine/Thymine/Cytosine, D = Guanine/Adenine/Thymine, H = Adenine/Cytosine/Thymine, V = Guanine/Cytosine/Adenine, N = Adenine/Guanine/Cytosine/Thymine).

To identify such features in promoter and *cis*-regulatory sequences amplified from selected *TaCKX* GFMs, we performed the motif conservation analysis using MEME Suite v5.4.1. The aim was to compare the *cis*-regulatory sequences, which were found in the three homologues of selected *TaCKX* GFMs based on the reference genome with that in our selected Kontesa and Ostka cvs. A conservation analysis of three maximum motif levels was performed. The *p*-value among all the motifs was recorded as 10^−44^ to 10^−77^, which shows that all the motifs discovered have a high confidence level and each of the motif widths was 50 base pairs, as shown in Figure 2. Comparative motif analysis among all reference genomes and our cvs. revealed that all sequences have at least three conserved motifs; however, there was a difference in their distribution and location level. For example, motifs 1-GSCGBYTWTMYATASGSKSYGCMMCYCYGAVGAGCAWMACAM-MAMWSGAY, 2-SCAGCAGMYMMTSRTMAYMRSATTARCRSRSCGAGSTAKSTARRC-GCG, and 3-GCVTCYHDKMGMVWTWMMGSMTRCGYWWMTGTTGSRMMGGSCGATCS-RTS of *TaCKX1-3A* (Figure 2A) in the reference genome are closely located compared to upstream sequences from the Kontesa and Ostka cvs. A similar trend of occurrence of motifs was observed in the upstream *cis*-regulatory sequences of *TaCKX2.2.1* in the Kontesa and Ostka cvs. compared to the reference genome sequences for three conserved motifs, i.e., motif 1- CAMMCYCAAGWMSTCKGTMGYRCCRYCTASMTASCTMRSTYKKCT, motif 2-YGCGTKKGW-KKCWTAGCTWGAGTRWTCKATVGWKYSAYCTM, and motif 3-YGCBSSSGTTWTTYMWWRSSCWCCVYCCSCMWGASRAGMRTCACAAAAAY (Figure 2B). However, motif 1 is located on the negative strand in the Kontesa and Ostka cvs., while motif 2 is located on the positive strand in the reference genome. Furthermore, three motifs of *TaCKX5* 1-CTTTAAGAATTCCGCTCCAAAGCTCGCCGCACATGGCACGCAGGATGAAA, 2-GAGTCGTGA-CGCGCCTCCGGATTTGTTTATTCTTGGGCGGCCCCAGCGGT, and 3-CGCGATCCACTAATTCT-GACAGGGGAATTAAGAGGCCGCCGGCCCGCGAC were found to be most conserved at base-pair level compared to the reference genome (Figure 2C).

The motifs of *TaCXK9*, i.e., 1-GAGGGATCAAGTGGGCCTGCTCGATAGCAATCAGGTTTGCCACTTGAATC, 2-GATTCCCCTT-TTCATGGAGTTCCCGGTTATCCACAGGATTGTCGCGTATG, and 3-CCATGTGGGCAAAGGGA-TTCTACAATATCAAGCTAAAGCTCCTGACCACT showed a pattern of motif conservation and distribution similar to *TaCKX5* (Figure 2D). Furthermore, among the three motifs of *TaCKX10*, i.e., motif 1-ATRMWTMTWTGAWTSTRTWTAGAMMAATAAMCAWWKAGGKSTT-CYGYRSA, motif 2-CSSCACY-GWYMKCYSBWCCCARCAGYTRMSCWGCMTKAATKRSGAR-TGGA, and motif 3-TRGSAAMACCCB-GSYSYTRCCSRMMTTWRTCRSCTYKHRGYRCATCSCCS (Figure 2E), motif 2 and 3 are located on the negative strand compared to the reference genome. The MEME suite GOMO tool v5.4.1 suggested that all selected motifs have a gene regulatory function.

### 2.4. Identification of TaNAC Transcription Factor Binding Sites in Selected TaCKX GFMs cis-Regulatory Sequences of Kontesa and Ostka cvs. and Selection of TaNAC Genes

However, the MEME suite provides information about the presence of common conserved motifs, to identify the TaNAC TFs and their binding sites on the *cis*-regulatory sequences of the selected *TaCKX* GFMs from the Kontesa and Ostka cvs. we again analyzed our sequences on the PlantTFDB v5.0 database. All parameters were the same as described in the previous section “Identification of TaNAC transcription factor binding sites of *TaCKX* GFMs *cis*-regulatory regions”. FIMO files containing details of all the transcription factors and their binding sites on the selected *TaCKX* GFMs are provided as Supplementary Folder S2. We identified and sorted the TaNAC transcription factors and their binding sites from the retrieved FIMO files and tabulated the results in Table 1. Based on the highest score and lowest *p*’ and q’ values of transcription factor binding sites in the *cis*-regulatory regions of each selected *TaCKX* GFM of the Kontesa and Ostka cvs., we selected one transcription factor per each basic *TaCKX* gene, which has been highlighted as blue in Table 2, for further analysis and characterization. Transcription factor binding site analysis of the selected *TaCKX* GFMs showed that TaNAC TF “Traes_5DL_0924913F8” is identified as the *transcription factor JUNGBRUNNEN 1-like [Triticum aestivum]* in the NCBI database. The TF binds to the “TACCGTCTTGATCCCGTC” sites of *TaCKX1-3A* gene *cis*-regulatory sites. Similarly, TaNAC TFs “Traes_1AL_C4FA8404A”, “Traes_2BL_209C14A8F”, “Traes_2BL_209C14A8F”, and “Traes_1AL_C4FA8404A” were identified in NCBI as NAC domain-containing protein 13-like [*Triticum aestivum*], putative NAC domain-containing protein 94 [*Triticum aestivum*], protein BEARSKIN1-like [*Triticum aestivum*], and NAC domain-containing protein 13-like [*Triticum aestivum*] and bind to the *cis*-regulatory sites “TGTAACTTGGGAGACAAGACA”, “TGCCGTATCTTGACCGGC”, “GGATGCTTAAAACATAAGCCA”, and “GAAAACTTGCTGATCACTACT” of the *TaCKX2.2.1-3B*, *TaCKX5-3D*, *TaCKX9-1B*, and *TaCKX10-7B* genes, respectively. In the case of *TaCKX2.2.1-3B* and *TaCKX10-7B*, a common TF (Traes_1AL_C4FA8404A) NAC domain-containing protein 13-like [*Triticum aestivum*] was identified to have binding interaction with the highest scores of 18.7576 and 12.7273, respectively. Therefore, for *TaCKX10-7B*, we also included the second TF “TRAES3BF002300010CFD_t1” identified as NAC domain-containing protein 48-like [*Triticum aestivum*] in NCBI for further analysis. The inclusion of a second TF for *TaCKX10-7B* allowed us to select at least one specific TaNAC for one selected *TaCKX* GFM, apart from the selection of common TaNACs involved in the regulation of more than one selected *TaCKX* genes.

### 2.5. NAC Domain Identification, Prediction of Protein Structure, and Various Attributes of Selected TaNACs

Although the plant transcription factor database provides a great deal of information about TFs such as TF ID, TF binding sites, the sites location on either positive or negative strands, and their start and end points on *cis*-regulatory sequences, various attributes such as TF gene ID, TF protein ID, gene description, and gene ontology are important features to fully characterize the transcription factors. We data-mined and retrieved the attributes of our selected TaNACs from different databases such as: PlantTFDB v5.0, EnsemblPlants v2.0, NCBI, QuickGo of EMBL-EBI, InterProScan, and SWISS-MODEL, and tabulated them (Table 2). The protein sequences of selected TaNACs were BLAST in the wheat EnsemblPlants v2.0 protein database and based on the highest identity index, the gene IDs were identified and the selected *TaNACs* namely *Transcription factor JUNGBRUNNEN 1-like*, *NAC domain-containing protein 13-like*, *putative NAC domain-containing protein 94*, and *protein BEARSKIN-1-like* were renamed to *TaNAC JUNGBRUNNEN-1* (*TaNACJ-1)*, *TaNAC13a*, *TaNAC94*, and *TaNAC BEARSKIN-1 (TaNACBr-1)*, respectively. Furthermore, the protein sequence of *NAC domain-containing protein 48-like* has a 100% identity index with *NAC 6D* so we renamed the *NAC domain-containing protein 48-like* to *TaNAC6D*. Gene ontology analysis performed on the QuickGo database showed that all of the selected TaNACs have DNA binding ability and that the nucleus is the site of a cellular compartment. Details of the gene ontology analysis of each of the selected *TaNAC* genes are listed in Table 2.

#### 2.5.1. Identification of the TaNAC Domain in Selected TaNAC Proteins

**Figure 3:**
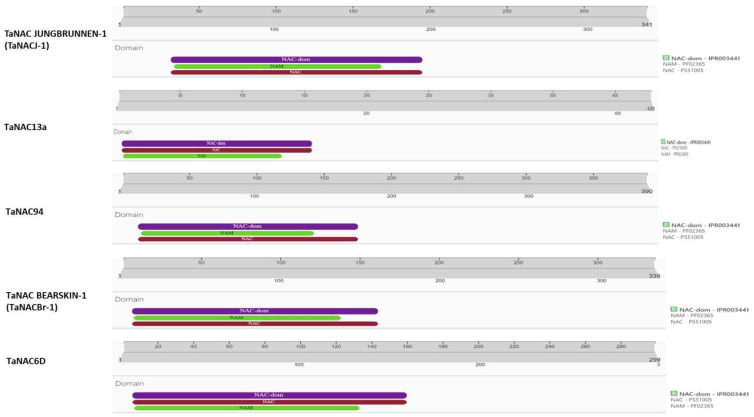
Identification of NAC domain in selected TaNACs by InterProScan database (accessed on 14 September 2023).

All selected TaNACs’ NCBI protein IDs were determined using transcription factor IDs. NCBI-retrieved protein sequences were analyzed for the presence of NAC domain confirmation using the InterProScan database. The analyzed file in the form of GFF was downloaded from Supplementary File S2. InterProScan analysis confirmed that TaNAC JUNGBRUNNEN-1 (TaNACJ-1), TaNAC13a, TaNAC94, TaNAC BEARSKIN-1 (TaNACBr-1), and TaNAC6D are 341 amino acid (aa), 429 aa, 390 aa, 336 aa, and 299 aa long proteins and have conserved NAC domains from 35 to 194 aa, 6 to 156 aa, 16 to 175 aa, 10 to 161 aa, and 9 and 159 aa, respectively, as show in Figure 3.

#### 2.5.2. Structure Prediction and Phylogenetic Analysis of Selected TaNACs

**Figure 4:**
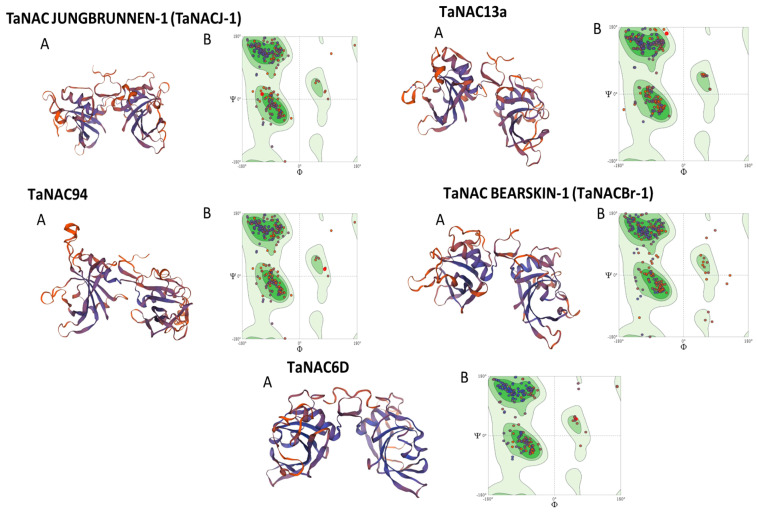
Tertiary structure prediction models of selected TaNAC proteins and Ramachandran plots. (**A**) Represents the tertiary structures of TaNAC JUNGBRUNNEN-1 (TaNACJ-1), TaNAC13a, TaNAC94, TaNAC BEARSKIN-1 (TaNACBr-1), and TaNAC6D (beta sheets in blue; alpha helixes in brown); (**B**) Ramachandran plots represent the residue in favored and unfavored regions (dots on the left in dark green circles represent residues in the favored regions, and dots on the right in lighter green circles represent residues in the unfavored regions).

To investigate the conservation of tertiary structure proteins of TaNAC JUNGBRUNNEN-1 (TaNACJ-1), TaNAC13a, TaNAC94, TaNAC BEARSKIN-1 (TaNACBr-1), and TaNAC6D, we modeled each selected TaNAC. The results indicated good structural quality with two alpha helixes and four beta sheets in each of the main domains of all selected TaNAC proteins (Figure 4A). The Ramachandran plots of the transcription factor JUNGBRUNNEN 1-like, NAC domain-containing protein 13-like, putative NAC domain-containing protein 94, protein BEARSKIN1-like, and NAC domain-containing protein 48-like indicated that the residues in the favored regions were 92.09%, 96.43%, 92.09%, 92.08%, and 94.12%, respectively (Figure 4B).

**Figure 5:**
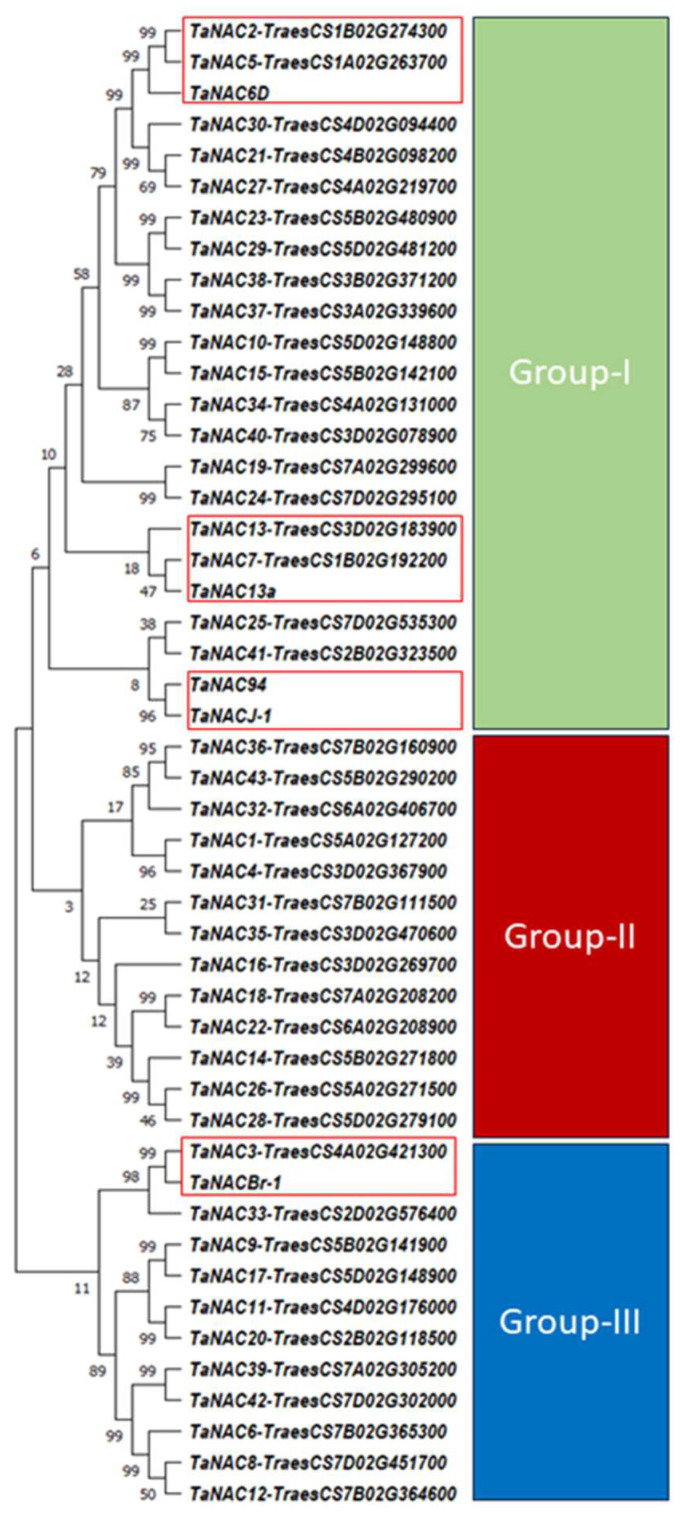
Maximum likelihood phylogenetic tree of TaNACJ-1, TaNAC13a, TaNAC94, TaNACBr-1, and TaNAC6D and 43 well-known TaNACs. The phylogenetic tree was generated using MEGA v.11 software, amino acid-based ClustlW-aligned sequences of TaNAC proteins. The selected TaNAC-containing clades are highlighted in redlined boxes.

To investigate the evolutionary relationship between 43 well-known TaNACs and TaNACJ-1, TaNAC13a, TaNAC94, TaNACBr-1, and TaNAC6D, phylogenetic analysis was performed by generating a maximum likelihood phylogenetic tree (File S3). The phylogenetic tree classified all TaNACs into three main groups. Four of our selected TaNACs, namely TaNAC6D, TaNAC13a, TaNACJ-1, and TaNAC94, were classified in group I. TaNAC6D shared a common clade with TaNAC2 and TaNAC5. TaNAC13a was in a single clade with TaNAC13 and TaNAC7; however, TaNACJ-1 and TaNAC94 were in a separate single clade showing a close evolutionary relationship between the two proteins. Furthermore, TaNACBr-1 was found in a common clade with TaNAC3 and TaNAC33 in group-III while none of the selected TaNACs were classified into group II (Figure 5).

### 2.6. Analysis of Expression of Selected TaNACs in Different Tissues of Developing Wheat Plants

**Figure 6:**
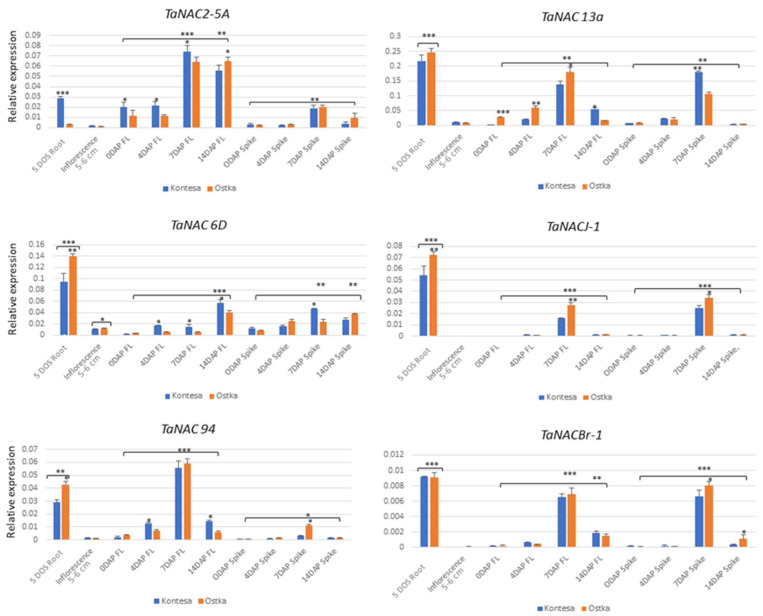
Specificity of expression of the selected *TaNAC* genes in various tissues of the Kontesa and Osktka cvs. Each bar is the mean value representation of three biological and three technical replicates. One-way and two-way ANOVAs were performed for statistical analysis. The asterisks above the bar indicate statistical significance compared to 0 DAP flag leaves and below the bar compared to Kontesa cv. (* 0.05 > *p* ≥ 0.01, ** 0.01 > *p* ≥ 0.001, *** *p* < 0.001).

The specificity of expression of *TaNACJ-1*, *TaNAC13a*, *TaNAC94*, *TaNACBr-1*, and *TaNAC6D* was analyzed in the various tissues of the Kontesa and Ostka cvs.: 5 DPG seedling roots, 5–6 cm long inflorescences, 0, 4, 7, and 14 DAP spikes, and the corresponding flag leaves. The expression data shown in Figure 6 are related to the expression of the *ADP Ribosylation factor-2* set as 1.00. For expression analysis, in addition to the selected *TaNACs*, we included *TaNAC2-5A* since our previous studies showed that this gene, could potentially be involved in coregulation of *TaCKX* GFMs [18,21].

Each selected *TaNAC* showed different gene expression patterns in the tested tissues. However, the level of expression in seedling roots of all tested *TaNACs* was high or very high compared to other tissues. The highest expression in the root was measured for *TaNAC13a* as 0.217 and 0.247 in the Kontesa and Ostka cvs., respectively. The lowest expression was measured for *TaNACBr-1* as 0.00914 and 0.00911 in the Kontesa and Ostka cvs., respectively. Furthermore, the expression of *TaNAC2-5A* in seedling roots was significantly higher in the Kontesa cv. (0.028) than the Ostka cv. (0.002). However, the expression of *TaNAC6D*, *TaNACJ-1*, and *TaNAC94* was significantly higher in the Ostka cv. with values of 0.139, 0.072, and 0.042 compared to the Kontesa cv. with values of 0.095, 0.054, and 0.028, respectively. There was no significant difference in the expression of *TaNACBr-1* in the roots of the Kontesa and Ostka cvs. (Figure 6).

The levels of expression of the tested *TaNACs* in inflorescences of the Kontesa and Ostka cvs. were very low compared to the other tissues tested. The highest expression was measured for *TaANC6D* in the Kontesa cv. as 0.010 and the Ostka cv. as 0.012, and the lowest expression was recorded in *TaNACBr-1* in the Kontesa cv. as 0.00005 and the Ostka cv. as 0.00009. The remaining expression levels of the selected *TaNACs*, including *TaNAC2-5A*, were restricted between the highest and lowest ranges in the Kontesa and Ostka cvs. (Figure 6).

In general, the expression of the selected *TaNACs* in the flag leaves and spikes gradually increased from 0 to 7 DAP and at 14 DAP it began to decline (Figure 6). The expression levels of *TaNAC2-5A* in the flag leaves were lowest at 0 DAP (0.019 in the Kontesa cv. and 0.011 in the Ostka cv.) but gradually increased in 7 DAP, reaching the values of 0.074 in the Kontesa cv and 0.064 in the Ostka cv. The expression at 14 DAP decreased to 0.055 in the Kontesa cv. and 0.064 in the Ostka cv. *TaNAC-5A* expression was higher and significantly different in the flag leaves of the Kontesa cv. compared to the Ostka cv. until 7 DAP; however, at 14 DAP the expression in the Ostka cv. remained higher than the Kontesa cv. and the difference was statistically significant. Similarly, *TaNAC2-5A* expression in the spikes followed the same pattern as that in the flag leaves from 0 DAP to 14 DAP. The highest expression of *TaNAC2-5A* in the spikes was at 7 DAP as 0.018 in the Kontesa cv. and 0.020 in the Ostka cv.

*TaNAC6D* showed the lowest expression in 0 DAP flag leaves as 0.0013 in the Kontesa cv. and 0.02 in the Ostka cv., while the highest expression in 7 DAP flag leaves as 0.138 and 0.179 in the Kontesa and Ostka cvs., respectively. The comparative expression pattern between the Kontesa and Ostka cvs. in the flag leaves revealed that, at 0 DAP, the expression of *TaNAC6D* was significantly higher in the Ostka cv. than in the Kontesa cv., and the expression pattern remained the same until 7 DAP between the two cvs. But, at 14 DAP, the expression of *TaNAC6D* in the Kontesa cv. was significantly higher than in the Ostka cv. However, in the spikes, *TaNAC6D* expression followed the same pattern as *TaNAC2-5A*. The lowest expression of *TaNAC6D* was recorded at 14 DAP as 0.0018 in the Kontesa cv. and 0.0043 in the Ostka cv., while the highest expression of *TaNAC6D* was recorded at 7 DAP as 0.180 in the Kontesa cv. and 0.106 in the Ostka cv. Unlike *TaANC2-5A* and *TaNAC13a*, the expression pattern of *TaNAC6D* in the flag leaves was slightly different. The expression of *TaNAC6D* in the flag leaves started from being the lowest at 0 DAP and reached a maximum at 14 DAP in both the Kontesa and Ostka cvs. (Figure 3). The lowest expression at 0 DAP was recorded as 0.0016 and 0.0036, while the highest expression at 14 DAP was recorded as 0.056 and 0.040 in the Kontesa and Ostka cvs., respectively. The expression of *TaNAC6D* in the spikes was highest at 7 DAP as 0.046 in the Kontesa cv., whereas at 14 DAP in the Ostka cv. it was 0.034.

*TaNACJ-1* exhibited a very distinctive 7 DAP specific expression pattern in flag leaves and spikes. The mean expression of *TaNACJ-1* was highest at 7 DAP in flag leaves (0.015 and 0.0278) and in spikes (0.025 and 0.039) in both Kontesa and Ostka cvs. correspondingly. However, Ostka cv. exhibited higher expression of *TaNACJ-1* in both tissues compared to Kontesa cv. *TaNAC94* and *TaNACBr-1* also exhibited a similar expression pattern to *TaNACJ-1* considering the 7 DAP time-dependent expression pattern in flag leaves and spikes, except for 14 DAP. *TaNAC94* maintained notable expression levels in flag leaves (0.014 and 0.006) in the Kontesa and Ostka cvs., respectively; meanwhile, *TaNACBr-1* expression at 14 DAP in the flag leaves was 0.0019 and 0.0015 in the Kontesa and Ostka cvs., correspondingly. The highest mean expression of *TaNAC 94* at 7 DAP was measured in the flag leaves (0.055 and 0.059) and in the spikes (0.003 and 0.01) for the Kontesa and Ostka cvs., respectively. Similarly, the highest expression of *TaNACBr-1* was observed at 7 DAP in the flag leaves (0.0065 and 0.0068) and in the spikes (0.0066 and 0.0080) in the Kontesa and Ostka cvs., respectively.

## 3. Discussion

### 3.1. TaNAC TFs Orchestrate cis-Regulation of TaCKX GFMs

The key role of *TaCKX* GFMs and *TaNAC* GFMs in controlling yield-related traits in wheat highlights the importance of exploring the TaNAC TF of *TaCKX* GFMs. To identify TaNAC TF binding sites on the *cis*-regulatory sequences of *TaCKX* GFMs, we initially performed an identification analysis in all 35 homologous genes of this family based on the reference wheat genome (IWGSC, RefSeq v2.1). The results showed that all of the *TaCKX* GFMs have TaNAC binding sites on their *cis*-regulatory sequences; however, each *TaCKX* showed different binding affinity parameters (File S1). This initial analysis helped us to choose only those *TaCKX* GFMs, which showed the highest scores and lowest values of “p” and “q” for TaNAC TFs binding to the *cis*-regulatory sites of *TaCKX* GFMs. We selected the five most important *TaCKX* GFMs, namely *TaCKX1-3A*, *TaCKX2.2.1-3B*, *TaCKX5-3D*, *TaCKX9-1B*, and *TaCKX10-7B* to further prove the identification of TaNAC TF binding sites in the selected *TaCKX* genes of the Kontesa and Ostka cvs.

Variability among the *cis*-regulatory sequences in the core promoters and distal promoter regions can change the gene regulation network and obstruct the evaluation of promoter efficiency [25]. Therefore, to identify such variability in the *cis*-regulatory sequences of the selected *TaCKX* GFMs compared to reference genome sequences from the Chinese Spring cv. (IWGSC, RefSeq v2.1) we first performed conserved motif MEME Suite analysis before identification of TaNAC TFs and their binding sites. The MEME Suite is an integrated online web server for the discovery and analysis of conserved motifs and identifies features such as DNA binding sites, their distance, distribution on positive or negative strands, and protein interaction domains [33]. The motif conservation analysis (Figure 2) showed that the motifs were conserved between the Kontesa and Ostka sequences compared to the reference genome sequences at the three motif levels; however, the distance and distribution patterns of the motif were different between the *TaCKX* GFMs between the Kontesa and Ostka cvs. compared to the reference genome (IWGSC, RefSeq v2.1). Knowing the spatial distribution, location, and pattern of the motifs is a subject of interest in steric stabilization, and such changes in *cis*-regulatory sequences lead to crop domestication [25,34].

After motif conservation analysis, we finally identified TaNAC TFs and their binding sites on the selected *TaCKX* GFMs in the Kontesa and Ostka cvs. Among the several TaNAC TFs that bind to the selected *TaCKX* GFM *cis*-regulatory sequences, we selected only one TaNAC TF with the highest binding affinity parameters for each selected *TaCKX* GFM, except *TaCKX10-7B* (Table 1), for further characterization. Similarly to us, in several studies, including Pompili et al. (2020), Yokotani et al. (2021) and Zhang et al. (2022) [35,36,37], other authors have used the plant transcription factor database (PlantTFDB v5.0) to identify transcription factors. The names of the selected TaNAC TFs in the NCBI database were identified as: transcription factor JUNGBRUNNEN 1-like, NAC domain-containing protein 13-like, putative NAC domain-containing protein 94, and protein BEARSKIN-1-like, which bind to the *cis*-regulatory sites of the *TaCKX1-3A*, *TaCKX2.2.1-3B*, *TaCKX5-3D*, *TaCKX9-1B*, and *TaCKX10-7B TaCKX* GFMs, respectively.

Interestingly, a deep analysis of TaNAC TFs that were listed in Table 1 revealed that some of them have common binding sites on more than one selected *TaCKX cis*-regulatory region and potentially are involved in their regulation. For example, putative NAC domain-containing protein 94 (Traes_2BL_209C14A8F) has a common binding site on *TaCKX5-3D* and *TaCKX1-3A* (Table 1 and Table 2). Similarly, the NAC domain-containing protein 13-like (Traes_1AL_C4FA8404A) showed common binding sites on the *cis*-regulatory regions of *TaCKX2.2.1-3B* and *TaCKX10-7B*, suggesting that the common TaNAC could coregulate more than one *TaCKX* gene.

### 3.2. Structural Characterization and Various Attributes Reveal the Recognizable Pattern of Selected TaNACs

When protein analysis is performed, the aim is always to find as much information as possible to explore its potential role and relationship with the other proteins. In the case of NAC TFs, all proteins contain a conserved NAC domain with the non-variable N-terminal and the variable C-terminal [38]. To identify conserved domains, several studies have performed domain identification analysis by scanning protein sequences in the InterProScan database [39,40]. Consistent with these studies, our underlined analysis of the NAC domain identified a conserved NAC domain in all our selected TaNAC TFs (Figure 3). The presence of this domain validated them as TaNAC TFs. Furthermore, their projected tertiary structure models are in agreement with the tertiary model predicted by Guérin et al. (2022) [41] for four TaGNAC TFs related to grain development. The phylogenic tree analysis classified all TaNAC TFs into three groups according to their potential role and the previously reported data (Figure 4). Mao et al. (2020) [42] identified eight drought-induced genes, i.e., *TaNAC30*, *TaNAC21*, *TaNAC27*, *TaNAC38*, *TaNAC10*, *TaNAC15*, *TaNAC34*, and *TaNAC40*. All of these genes were present in group I with four of our selected *TaNACs*, namely *TaNAC6D*, *TaNAC13a*, *TaNACJ-1*, and *TaNAC94*, suggesting their potential role in drought stress. As investigated by us, *TaNAC2-5A* was also classified into group I. *TaNAC2-5A* is a gene encoding nitrate-inducible TaNAC TF, well characterized for its role in root growth, grain yield, soil nitrate uptake, establishment of seed vigor, and drought stress [31,32,43]. Furthermore, our selected *TaNACBr-1* was classified into group III of the phylogenetic tree, which contains TaNAC TFs with a potential role in responses to drought, heat, and salt stress [44,45]. Tang et al. (2011) [46], in their study on the tolerance to drought stress conferred by *TaNAC* GFMs, classified *TaNAC13* and *TaNAC7* as development-related. Therefore, the potential role of our selected *TaNAC13a*, which shared the same common clade with *TaNAC13* and *TaNAC7*, might also be associated with plant development. Two genes responsive to biotic stress, *TaNAC30* and *TaNAC35*, that have been known to regulate leaf rust stress in wheat [47,48], were classified in group II of the phylogenetic tree.

The Gene Ontology (GO) resource is used to explain various aspects such as biological processes, molecular functions, and cellular localization of a gene product [49]. GO analysis showed that of all the selected *TaNAC* GFMs have transcription-regulation and DNA template-binding ability as their common biological and molecular functions, and the nucleus as their cellular compartment, which confirms them as transcription factors. Furthermore, the GO analysis of two of the selected *TaNACs* (*TaNAC94* and *TaNACBr-1*) showed their potential role in the development of the root cap, while *TaNACJ-1* and *TaNAC6D* showed their potential role in the negative regulation of leaf senescence and the regulation of the abscisic acid-activated signaling pathway, respectively (Table 2). Other important biological processes regulated by the selected *TaNACs* are listed in Table 2.

### 3.3. Selected TaNACs Are Preferentially Expressed in the Roots

The root is an important organ in plants that is not only responsible for the regrowth of the plant in the soil but also serves to absorb water and nutrients from the soil [50,51]. In wheat crops, several TaNAC TFs have been reported to regulate the root system. Overexpression of *TaRNAC1* positively regulated the root system and increased above-ground biomass, while overexpression of *TaSNAC8-6A* helped with lateral root system development by activating drought-responsive and auxin-signaling genes [42]. Conversely, *TaNAC14* overexpression negatively regulated root growth [52]. The *TaNAC* GFMs selected in this investigation were preferentially expressed in the roots of two cultivars, suggesting their potential role in root growth and development (Figure 6). Our expression analysis is in agreement with the GO analysis, where *TaNACJ-1* showed the potential role in the response to hyperosmotic salinity, while *TaNAC94* and *TaNACBr-1* showed a direct role in root cap regulation (Table 2). *TaCKX1* and *TaCKX5* have been shown to be highly expressed in seedling roots [22] and the TF identification results of this study (Table 2) have shown that TaNAC94 TF is potentially involved in *cis*-regulation of both *TaCKX1* and *TaCKX5* GFMs. The preferential expression of *TaNAC94* in seedling roots strongly suggests the potential role of TaNAC94 TF in the coregulation of *TaCKX1* and *TaCKX5*. As drought is a serious challenge to crop productivity [53], the identification of gene functions associated with root growth and development is considered one of the best ways to improve crop productivity. Since all of our selected *TaNACs* showed expression specificity to the root, further characterization of these selected *TaNACs* could be beneficial in crop productivity.

### 3.4. Inflorescence Is Not the Primary Site for the Expression of Our Selected TaNAC GFMs

Inflorescence is the arrangement of the flowers during the flowering process [54]. This is an important part of the plant in which to assess the expression level of genes related to reproductive parts during flowering time. Some *TaNAC* GFMs, such as *TaNACS-1A*, have already been reported to be preferably expressed during the flowering days in wheat [55,56], while *TaNAC8* was less favorable in the flowers and stem compared to the developing seeds [57]. We found that the selected *TaNACs* do not express preferably in inflorescence, except *TaNAC6D* (Figure 6).

### 3.5. The Selected TaNACs Showed a Distinct Preference for Expression in Flag Leaves and Spikes during the 7 and 14 DAP Periods

*TaNAC* genes related to seed-associated traits directly influence crop productivity, while *TaNAC* GFMs related to leaves or leaf senescence indirectly influence crop productivity [25]. We explored the expression pattern of the selected *TaNACs* in the spikes and flag leaves of the two cultivars collected during four developmental stages: 0, 4, 7, and 14 DAP. Each selected *TaNAC* showed a discrete pattern of expression; however, at 7 and 14 DAP, the level of expression was higher compared to the 0 and 4 DAP. It is notable to mention that, at some developmental stages, we observed different levels of expression of our selected *TaNAC* GFMs between the Kontesa and Ostka cvs., especially in the flag leaves and spikes (Figure 6). These differences in expression suggest that it can be related to the spike architecture awned (Ostka) or awnless (Kontesa) cultivars [18]. It has already been shown that 7 and 14 DAP spikes are sites of preference for some of the *TaCKX* GFMs. For example, *TaCKX1* and the group of *TaCKX2* genes were preferentially expressed at 7 and 14 DAP in the spikes of the Kontesa, Ostka, and Trappe cvs. [22]. The highest expression in 7 DAP spikes of 34 breeding lines has also been observed for *TaCKX1*, *TaCKX11*, and *TaNAC2-5A* [21]. Our results showed that the selected *TaNAC* GFMs, including *TaNACJ-1*, *TaNAC13a*, *TaNAC94*, *TaNACBr-1*, and *TaNAC6D*, interact with the *cis*-regulatory sites of *TaCKX1-3A*, *TaCKX22.1-3B*, *TaCKX5-3D*, *TaCKX9-1B*, and *TaCKX10*, respectively, and potentially regulate their expression in the spikes and flag leaves. *TaNACs* have been known to regulate seed-related traits such as seed germination, seed vigor, and seed storage protein [5,23,28]. The significance of the spike-related expression of our selected *TaNACs* may be correlated to such grain-related traits. Similarly, the leaf senescence trait has been reported to be regulated by various *TaNACs*, including *TaNAC29*, *NAM-A1*, and *TaSNAC11-4B* [58,59,60]. The preferential expression of our selected *TaNACs* in the flag leaves also suggests their potential role in leaf senescence.

## 4. Materials and Methods

### 4.1. Acquisition of TaCKX GFMs’ cis-Regulatory Sequences and Identification of TaNAC Transcription Factor Binding Sites

For the acquisition of 1.5 kb upstream promoter and *cis*-regulatory sequences including UTRs of all *TaCKX* GFMs, the wheat EnsemblPlants database (https://plants.ensembl.org/Triticum_aestivum/Info/Index accessed on 22 June 2022), RefSeq v2.1 of the International Wheat Genome Sequencing Consortium (IWGSC) was used [61,62]. The acquired sequences were used to identify TaNAC transcription factor binding sites in these sequences using the PlantTFDB v5.0 online web server database (http://planttfdb.gao-lab.org/ accessed on 4 July 2022) [63]. Find Individual Motif Occurrence (FIMO) files were downloaded from the PlantTFDB v5.0 database for each homolog of the selected *TaCKX* GFMs containing the details of all the transcription factor binding sites. From these FIMO files, TaNAC transcription factor binding sites were identified.

### 4.2. Plant Materials and Growth Conditions

Two Polish spring wheat cultivars, Kontesa and Ostka, were used in the experiments. The seeds of both cultivars were grown in Petri dishes at 4 °C for one day and at room temperature (22 °C) in dark conditions for four days. The seedlings were then replanted in pots containing peat soil and grown in the growth chamber with the following conditions: 20 °C/18 °C day/night temperature, 16/8 h light/dark photoperiod, 350 µmol m^−2^ s^−1^ light intensity, and 70% humidity. Four-week-old fresh leaves were selected for DNA extraction, and the samples were frozen in liquid nitrogen prior to DNA extraction.

### 4.3. Genomic DNA Extraction and PCR Amplification

Genomic DNA was extracted from young leaf samples from the Kontesa and Ostka cvs. using the CTAB method as described by Yu et al. (2017) [64]. Nanodrop (NanoDrop ND-1000) was used to measure concentration and purification, and 0.8% agarose gel was used to verify DNA integrity. The primers were designed using the DNASTART SeqMAN pro primer design option (Table S1) to amplify targeted cis-regulatory sequences for the selected TaCKX GFMs. The conditions for the PCR reaction were optimized as: initial denaturation and polymerase activation at 98 °C for 3 min, (98 °C for 30 s, 60 °C for 30 s, 72 °C for 1.5 min) × 35 cycles, and the final extension at 72 °C for 5 min. High-fidelity Phusion DNA polymerase (Thermo Fisher Scientific, Waltham, MA, USA) was used for PCR.

### 4.4. Cloning and Sequencing of Selected TaCKX cis-Regulatory Sequences of the Kontesa and Ostka Cultivars

The PCR amplicons of selected *TaCKX* GFM *cis*-regulatory regions were subjected to PCR purification using E.Z.N.A. Cycle Pure Kit (Omega Bio-TEK Inc, Norcross, GA, USA) and quantified using Nanodrop (NanoDrop ND-1000, Thermo Fisher Scientific, Wilmington, DE, USA). The blunt ends of the PCR amplicons produced by Phusion DNA polymerase were A ‘tailed’ using the HighTaq DNA polymerase kit (BIORON GmbH, Römerberg, Germany). The reaction mixture contained: 6 μL of PCR amplicon, 1 μL of HighTaq DNA polymerase 10× reaction buffer, 0.2 μL of 10 mM dATP, 1 μL of HighTaq polymerase (5 U/μL), and 0.8 μL of deionized water. A total of 10 μL of the reaction was incubated at 70 °C for 30 min and 1 μL was used for ligation to the pGEMT easy vector (Promega, Madison, WI, USA) following the manufacturer’s instructions. The DH5α strain of *E. coli* was used to prepare competent cells following the method reported by Chung et al. (1989) [65] with few modifications, and pGEMT easy recombinant vectors were transformed into DH5α competent cells using the BIO-RAD GENE Pulser apparatus at 25 microfarad (µF) capacitance and 2.5 kv voltage. Positive colonies were confirmed by the PCR amplification method and were sequenced from GENOMED (https://www.genomed.pl/).

### 4.5. Motif Conservation Analysis

For the analysis of homology and conservation of motifs of the upstream *cis*-regulatory region of each selected *TaCKX* GFM, the MEME Suite v5.4.1 (Introduction—MEME Suite) (meme-suite.org) database was used. A single text file was generated containing each selected *TaCKX* gene and its homologous sequences from the reference genome and selected sequences of the Kontesa and Ostka cvs. and was uploaded to the MEME suite for motif conservation. A maximum of three numbers of motifs were selected for conservation analysis.

### 4.6. Identification of TaNAC Transcription Factor Binding Sites in Selected TaCKX GFMs

The promoter and *cis*-regulatory sequenced regions of selected *TaCKX* GFM from the Kontesa and Ostka cvs. were analyzed in the PlantTFDB v5.0 database to identify TaNAC binding sites and their corresponding TaNAC TFs.

### 4.7. NAC Domain Identification, Prediction of Protein Structure, and Phylogenetic Analysis

The protein sequences of the transcription factor JUNGBRUNNEN 1-like, NAC domain-containing protein 13-like, putative NAC domain-containing protein 94, protein BEARSKIN1-like, and NAC domain-containing protein 48-like were scanned in the InterProScan database (https://www.ebi.ac.uk/interpro/ accessed on 14 September 2023) to identify and confirm the presence of NAC domains in the selected TaNACs. For protein structure prediction and validation, SWISS-MODEL (https://swissmodel.expasy.org/ accessed on 18 October 2023) was used. Phylogenetic tree analysis was performed using MEGA v.11 software.

### 4.8. RNA Extraction and cDNA Synthesis

For expression analysis, the following tissue samples were collected: 5-day-old seedling roots, 5–6 cm long inflorescences, 0, 4, 7 and 14 days after pollination (DAP) spikes and accompanying flag leaves. All samples were frozen in liquid nitrogen prior to RNA extraction. Each sample was collected in three biological replicates, and each biological sample was measured in three technical replicates. Total RNA was extracted from all selected tissues and different time intervals using TRIzol RNA extraction reagent (Thermo Fisher Scientific) following the manufacturer’s protocol. RNA was quantified by Nanodrop (NanoDrop ND-1000) and integrity was checked by running 2 μL of RNA on a 2% agarose gel. The RNA was treated with DNase I to remove DNA contamination before proceeding with the synthesis of cDNA. The RevertAid First Strand cDNA Synthesis Kit (Thermo Fisher Scientific) was used to reverse transcribe 1 μg of RNA into cDNA following the manufacturer’s protocol. The 1 μL cDNA was further diluted to 20 μL for the final concentration to be used for RT-qPCR.

### 4.9. Quantitative RT-qPCR

Six TaNAC genes were subjected to the RT-qPCR assay (TaNAC94-like: XM_044560882.1, TaNAC JUNGBRUNNEN 1-like: XM_044546100.1, TaNAC13-like: XM_044594559.1, TaNAC48-like: XM_044503013.1, and TaNAC BEARSKIN1-like: XM_044472340: TaNAC2-5A AY625683). The primers were designed using the DNASTART SeqMAN pro Primer design option (Table S2) to amplify each selected gene. All RT-qPCR reactions were performed on iQ5 Cycler (BIO-RAD, Hercules, CA, USA) as described by Ogonowska et al. (2019) [22]. The PCR profile was followed as: initial denaturation and polymerase activation at 95 °C for 3 min, (95 °C for 20 s, 63 °C for 67 °C 20 s, 72 °C for 25 s) × 45 cycles, final extension at 72 °C for 5 min, and melting curve from 65 to 95 °C for 25 s per degree Celsius of increment. Each value is the mean representation of the three biological and three technical replicates. Ref-2 (*ADP ribosylation factor 2*) was used as a normalizer (internal control) and a comparative control for the relative expression of selected *TaNACs* in a two-standard curve method of RT-qPCR.

### 4.10. Statistical Analysis

For statistical analysis, one-way and two-way analysis of variance (ANOVA) tests were performed to determine the level of significant differences between the expression levels of the Kontesa and Ostka cvs. GraphPad Prism v8.0 software for Windows was used to perform these statistical data analysis tests. Data bars with statistically significant differences and having *p*-values ≤ 0.05, 0.01 and 0.001 were indicated by asterisks as *, **, and ***, respectively.

## 5. Conclusions

NAC transcription factors are one of the largest TF families in plants, and *TaNAC GFMs* have been known to regulate several physiological processes in bread wheat by interacting with sequences on promoters or upstream *cis*-regulatory sites. Similarly, *TaCKX* family members are important genes that encode CKX enzyme to regulate cytokinin concentration by their degradation and consequently influence wheat yield-related traits. Therefore, in this study, we focused on identifying important *TaNAC* genes that could be involved in the *cis*-regulation of *TaCKX* GFMs. These results provide useful information to further characterize these selected *TaNAC* GFMs for their role in root growth and development, grain productivity, leaf senescence, and response to various stresses.

## Figures and Tables

**Table 1:** Summary of identified TaNAC transcription factors and their binding sites in *cis*-regulatory regions of selected *TaCKX* GFMs.

Selected *Ta*CKX	TaNAC TF ID	Family	Start	Stop	Strand	Score	*p*-Value	q-Value	Transcription Factor Binding Sites
*TaCKX1* (Threshold *p*-value 10^−4^)									
*TaCKX1-3A* (TraesCS3A02G109500)									
	**Traes_5DL_0924913F8**	**NAC**	**552**	**571**	**+**	**14.4721**	**3.05 × 10^5^**	**0.0668**	**TACCGTCTTGATCCCGTC**
	Traes_6BL_A169A3ECB	NAC	707	724	+	14.1667	**1.04 × 10^5^**	0.0402	TACCGTCTTGATCCCGTC
	Traes_6AL_0D7866801	NAC	707	724	+	14.1667	**1.04 × 10^5^**	0.0402	TACCGTCTTGATCCCGTC
	TRAES3BF114300070CFD_t1	NAC	1492	1510	−	11.8333	**4.54 × 10^5^**	0.173	CTTCTCCGGTAGGGCACCC
*TaCKX2.2.1* (Threshold *p*-value 10^−4^)									
*TaCKX2.2.1-3B* (TraesCS3B02G161000)									
	**Traes_1AL_C4FA8404A**	**NAC**	**1113**	**1133**	**+**	**18.7576**	**3.38 × 10^7^**	**0.00133**	**TGTAACTTGGGAGACAAGACA**
	Traes_4BL_D592DBAAF	NAC	1115	1130	−	16.4545	**1.45 × 10^6^**	0.00573	CTTGTCTCCCAAGTTA
	Traes_4AS_B95A1C3A1	NAC	1115	1135	+	15.8636	**2.57 × 10^6^**	0.0101	TAACTTGGGAGACAAGACATT
	Traes_1AL_C4FA8404A	NAC	1115	1135	−	14.3182	**4.29 × 10^6^**	0.00848	AATGTCTTGTCTCCCAAGTTA
	Traes_4BS_373BDBA94	NAC	1115	1130	−	14.9242	**5.50 × 10^6^**	0.0218	CTTGTCTCCCAAGTTA
	Traes_5BL_657BF1497	NAC	1115	1132	+	14.4394	**6.70 × 10^6^**	0.0265	TAACTTGGGAGACAAGAC
	Traes_1BL_8925B27BC	NAC	1116	1130	+	14.3333	**8.20 × 10^6^**	0.0325	AACTTGGGAGACAAG
	Traes_7AL_46F73E667	NAC	1113	1133	+	13.7727	**9.26 × 10^6^**	0.0366	TGTAACTTGGGAGACAAGACA
	Traes_2BL_209C14A8F	NAC	1113	1133	+	12.6061	**1.61 × 10^5^**	0.0636	TGTAACTTGGGAGACAAGACA
	Traes_4AS_B95A1C3A1	NAC	1113	1133	−	11.7273	**1.84 × 10^5^**	0.0364	TGTCTTGTCTCCCAAGTTACA
	Traes_4BS_373BDBA94	NAC	1118	1133	+	12.5	**2.39 × 10^5^**	0.0474	CTTGGGAGACAAGACA
	Traes_5DL_B69423A67	NAC	1116	1132	+	12.7121	**2.47 × 10^5^**	0.0979	AACTTGGGAGACAAGAC
	Traes_4AL_99942CBEA	NAC	1115	1131	+	12.0758	**3.72 × 10^5^**	0.147	TAACTTGGGAGACAAGA
*TaCKX5* (Threshold *p*-value 10^−4^)									
*TaCKX5-3D* (TraesCS3D02G310200)									
	**Traes_6BL_A169A3ECB**	**NAC**	**1213**	**1230**	**−**	**14.2273**	**9.92 × 10^6^**	**0.0386**	**TGCCGTATCTTGACCGGC**
	Traes_6AL_0D7866801	NAC	1213	1230	−	14.2273	**9.92 × 10^6^**	0.0386	TGCCGTATCTTGACCGGC
	Traes_5DL_0924913F8	NAC	1211	1230	−	13.7727	**1.16 × 10^5^**	0.0445	TGCCGTATCTTGACCGGCTT
	Traes_5BL_39FF03C5D	NAC	1211	1230	−	13.7727	**1.16 × 10^5^**	0.0445	TGCCGTATCTTGACCGGCTT
*TaCKX9* (Threshold *p*-value 10^−4^)									
*TaCKX9-1B* (TraesCS1B02G248700)									
	**Traes_2BL_209C14A8F**	**NAC**	**1197**	**1217**	**+**	**16.4394**	**1.71 × 10^6^**	**0.00463**	**GGATGCTTAAAACATAAGCCA**
	Traes_7AL_46F73E667	NAC	1197	1217	+	16.2424	**1.94 × 10^6^**	0.00536	GGATGCTTAAAACATAAGCCA
	Traes_4AL_99942CBEA	NAC	1199	1215	+	14.8939	**5.19 × 10^6^**	0.0142	ATGCTTAAAACATAAGC
	Traes_4AL_99942CBEA	NAC	1201	1217	−	14.0606	**1.01 × 10^5^**	0.0142	TGGCTTATGTTTTAAGC
	Traes_2AL_82C3E7E14	NAC	1200	1214	−	13.3485	**1.80 × 10^5^**	0.0328	CTTATGTTTTAAGCA
	Traes_6AL_8BA1FF8B2	NAC	882	893	−	11.798	**2.03 × 10^5^**	0.062	GGACAAGCCAAG
	Traes_2AL_82C3E7E14	NAC	1202	1216	+	12.9848	**2.32 × 10^5^**	0.0328	CTTAAAACATAAGCC
*TaCKX10* (Threshold *p*-value 10^−4^)									
*TaCKX10-7B* (TraesCS7B02G264400)									
	**Traes_1AL_C4FA8404A**	**NAC**	**468**	**488**	**+**	**12.7273**	**9.05 × 10^6^**	**0.0318**	**GAAAACTTGCTGATCACTACT**
	**TRAES3BF002300010CFD_t1**	**NAC**	**406**	**427**	**−**	**12.5152**	**2.33 × 10^5^**	**0.0856**	**TTCGTGTTTGTATTGGCCACGT**
	TRAES3BF114300070CFD_t1	NAC	160	178	+	12.2576	**3.46 × 10^5^**	0.133	CCTGTATTTCACGGAGTCG
	Traes_5DL_0924913F8	NAC	1023	1042	−	11.5455	**4.64 × 10^5^**	0.178	CTCCGTTTTATTTACTCCGC
	Traes_5BL_39FF03C5D	NAC	1023	1042	−	11.5455	**4.64 × 10^5^**	0.178	CTCCGTTTTATTTACTCCGC
	Traes_2DS_5DF921ABB	NAC	1527	1534	−	12.0303	**6.67 × 10^5^**	0.258	TACGTAAT
* TaCKX2.1 **(Threshold *p*-value 10^−4^)**									
*TaCKX2.1-3D* TraesCS3D02G143600									
	**Traes_5DL_0924913F8**	**NAC**	**653**	**672**	**+**	**12.2727**	**3.05 × 10^5^**	**0.0668**	**CGTCGTGCTCATCCCGGAGC**
	Traes_6BL_A169A3ECB	NAC	653	670	+	12.5152	**3.10 × 10^5^**	0.0952	CGTCGTGCTCATCCCGGA
	Traes_5DL_0924913F8	NAC	537	556	+	11.697	**4.26 × 10^5^**	0.0668	CGGCGTGGGCGTCAGGGCAC
* TaCKX11 **(Threshold *p*-value 10^−4^)**									
*TaCKX11-7B* TraesCS7B02G455000									
	**Traes_2BL_209C14A8F**	**NAC**	**687**	**707**	**−**	**9.48485**	**6.29 × 10^5^**	**0.115**	**CATAAATTCAAATTTAATCAA**
	Traes_2BL_209C14A8F	NAC	685	705	+	8.98485	**7.61 × 10^5^**	0.115	AATTGATTAAATTTGAATTTA

* according to sequences from the EnsemblPlants database, RefSeq v2.1.

**Table 2:** Summary of various attributes of selected *TaNAC* genes.

Attributes	Selected *TaNACs*				
	Transcription Factor JUNGBRUNNEN 1-like [*Triticum aestivum*]	NAC Domain-Containing Protein 13-like [*Triticum aestivum*]	Putative NAC Domain-Containing Protein 94 [*Triticum aestivum*]	Protein BEARSKIN1-like [*Triticum aestivum*]	NAC Domain-Containing Protein 48-like [*Triticum aestivum*]
***TaCKX*** **GFMs Regulated by TaNACs**	*TaCKX1-3A* TraesCS3A02G109500 *TaCKX2.1-3D* TraesCS3D02G143600 *TaCKX5-3D* TraesCS3D02G310200	*TaCKX 2.2.1-3B* TraesCS3B02G161000 *TaCKX10-7B* TraesCS7B02G264400	*TaCKX5-3D* TraesCS3D02G310200 *TaCKX1-3A* TraesCS3A02G109500 *TaCKX2.1-3D* TraesCS3D02G143600	*TaCKX9-1B* TraesCS1B02G248700 *TaCKX 2.2.1-3B* TraesCS3B02G161000 *TaCKX11-7B* TraesCS7B02G455000	*TaCKX10-7B* TraesCS7B02G264400
**Rename of selected *TaNACs***	*TaNAC JUNGBRUNNEN-1 (TaNAC J-1)*	*TaNAC13a*	*TaNAC94*	*TaNAC BEARSKIN-1* *(TaNAC Br-1)*	*TaNAC6D*
**TaNAC TF ID**	Traes_5DL_0924913F8	Traes_1AL_C4FA8404A	Traes_6BL_A169A3ECB	Traes_2BL_209C14A8F	TRAES3BF002300010CFD_t1
**TaNAC Protein ID**	XP_044402035.1	XP_044450494.1	XP_044416817.1	XP_044328275.1	XP_044358948.1
**Protein size**	341 aa	429 aa	390 aa	336 aa	299 aa
**NAC domain size**	35-194 aa	6-156 aa	16-175 aa	10-161 aa	9-159 aa
**NCBI Gene ID**	XM_044546100.1	XM_044594559.1	XM_044560882.1	XM_044472340	XM_044503013.1
**Ensemble Gene ID**	TraesCS5D02G421100	TraesCS1D02G266500	TraesCS6D02G286300	TraesCS2D02G309800	TraesCS3D02G401200
**Gene Description at Plant Ensemble**	n/a	n/a	n/a	n/a	NAC6D
**Gene Ontology Analysis**	➢ Regulation of transcription, DNA-templated ➢ Trehalose biosynthetic process ➢ Proline biosynthetic process ➢ Anthocyanin-containing compound biosynthetic process ➢ Camalexin biosynthetic process ➢ Hyperosmotic salinity response ➢ Negative regulation of leaf senescence ➢ Cellular component-nucleus ➢ Molecular function-DNA binding	➢ Regulation of transcription, DNA-templated ➢ Cellular component-nucleus ➢ Molecular function-DNA binding	➢ Regulation of transcription, DNA-templated ➢ Response to auxin ➢ Positive regulation of asymmetric cell division ➢ Somatic stem cell division ➢ Root cap development ➢ Cellular component-nucleus ➢ Molecular function-DNA binding	➢ Regulation of transcription, DNA-templated ➢ Plant-type secondary cell wall biogenesis ➢ Root cap development ➢ Cellular component-nucleus ➢ Molecular function-DNA binding	➢ Regulation of transcription, DNA-templated ➢ Response to wounding ➢ Negative regulation of abscisic acid-activated signaling pathway ➢ Cellular component-nucleus ➢ Molecular function-DNA binding

## Data Availability

All data used/generated in this study have been included in the text and Supplementary Materials of this manuscript.

## Supplementary Materials

The following supporting information can be downloaded at: https://www.mdpi.com/article/10.3390/ijms25042027/s1.
