# Supplementary material for: Are TaNAC Transcription Factors Involved in Promoting Wheat Yield by cis-Regulation of TaCKX Gene Family?
Source: Int J Mol Sci. 2024 Feb 7;25(4):2027. doi: 10.3390/ijms25042027 (PMC10889182; doi:10.3390/ijms25042027)
Supplement: Supplementary file 1 [file ijms-25-02027-s001.zip › Table S1.pdf]

Gene	Forward	Reverse
**CKX1-3A**	CGTGTGATATATACCAAACATAGGTACG	TAGCGCCTCGCGTTTAGCT
**CKX2.2.1-3B**	TGCACTCCCCTCGTGATACTAAT	CTCCTGCGTGTGTGTCTTAGC
**CKX5-3D**	GAATCAAATGACACTCTTTTCTATCA	TGCCACTCTCTGTCTACCTCTCT
**TaCKX9-1B**	CACTTGGTGGAATCACTAGAAAGA	CAGGGTAGTGGTTCACTGCAAG
**CKX10-7B**	ACTCATCTAGAAACACGTGGCC	AGCTTGAGTTTGCTACTTGCA
