# Supplementary material for: Are TaNAC Transcription Factors Involved in Promoting Wheat Yield by cis-Regulation of TaCKX Gene Family?
Source: Int J Mol Sci. 2024 Feb 7;25(4):2027. doi: 10.3390/ijms25042027 (PMC10889182; doi:10.3390/ijms25042027)
Supplement: Supplementary file 1 [file ijms-25-02027-s001.zip › Table S2.pdf]

Gene	Forward	Reverse
**TaNAC2-5A**	CAAGTACCCCAACGGCTCC	AGTCGGTCTTGACCCCCTTA
**TaNAC-13a**	TTCTCCTCCAGCTTTCGGTT	ACAGAATTCAGGGCCACAGA
**TaNAC-48**	GGCGAGAAGGAGTGGTACTT	CTCGTGCATGATCCAGTTGG
**TaNAC-94**	ACGCCTCAGAGAACACATCA	GCGGGATTGCTGAGAGGATA
**TaNAC JUNGBRUNNEN 1**	GCGGCAACTTCTTCTACGAG	ATCGGTGACCTCCATCATCC
**TaNAC Bearskin-1**	TCCTGAAAATACTGGTTGGAGAGT	GCAAACACATGTACACGCACAAA
