# Supplementary material for: Are TaNAC Transcription Factors Involved in Promoting Wheat Yield by cis-Regulation of TaCKX Gene Family?
Source: Int J Mol Sci. 2024 Feb 7;25(4):2027. doi: 10.3390/ijms25042027 (PMC10889182; doi:10.3390/ijms25042027)
Supplement: Supplementary file 1 [file ijms-25-02027-s001.zip › Table S3.pdf]

Table S3: ***TaCKX*** **GFMs and their homolog IDs. Selected genes are highlighted in bold.**

Sr.#	*TaCKX Basic Genes*	*Gene Homoeologs*	*Gene IDs*
1	***TaCKX1***	***TaCKX1-3A***	***TraesCS3A02G109500***
		TaCKX1-3B	*TraesCS3B02G128700*
		TaCKX1-3D	*TraesCS3D02G111300*
2	*TaCKX2.1*	*TaCKX2.1-3A*	*TraesCS3A02G311000*
		TaCKX2.1-3B	*TraesCS3B02G161100*
		TaCKX2.1-3D	*TraesCS3D02G143600*
3	***TaCKX2.2.1***	*TaCKX2.2.1-3A*	*TraesCS3A02G311100*
		TaCKX2.2.1-3B	***TraesCS3B02G161000***
		TaCKX2.2.1-3D	*TraesCS3D02G143500*
4	*TaCKX2.2.2*	*TaCKX2.2.2-3D*	*TraesCS3D02G143300*
5	*TaCKX2.2.3*	*TaCKX2.2.3-3D*	*TraesCS3D02G143200*
6	*TaCKX3*	*TaCKX3-1A*	*TraesCS1A02G159600*
		TaCKX3-1B	*TraesCS1B02G176000*
		TaCKX3-1D	*TraesCS1D02G157000*
7	*TaCKX4*	*TaCKX4-3A*	*TraesCS3A02G481000*
		TaCKX4-3B	*TraesCS3B02G525300*
		TaCKX4-3D	*TraesCS3D02G475800*
8	***TaCKX5***	*TaCKX5-3A*	*TraesCS3A02G321100*
		TaCKX5-3B	*TraesCS3B02G344600*
		TaCKX5-3D	***TraesCS3D02G310200***
9	*TaCKX7*	*TaCKX7-6A*	*TraesCS6A02G185800*
		TaCKX7-6B	*TraesCS6B02G214700*
		TaCKX7-6D	*TraesCS6D02G172900*
10	*TaCKX8*	*TaCKX8-2A*	*TraesCS2A02G378300*
		TaCKX8-2B	*TraesCS2B02G395200*
		TaCKX8-2D	*TraesCS2D02G374600*
11	***TaCKX9***	*TaCKX9-1A*	*TraesCS1A02G234800*
		TaCKX9-1B	***TraesCS1B02G248700***
		TaCKX9-1D	*TraesCS1D02G237200*

12	***TaCKX10***	*TaCKX10-7A*	*TraesCS7A02G363400*
		TaCKX10-7B	***TraesCS7B02G264400***
		TaCKX10-7D	*TraesCS7D02G359700*
13	*TaCKX11*	*TaCKX11-7A*	*TraesCS7A02G536900*
		TaCKX11-7B	*TraesCS7B02G455000*
		TaCKX11-7D	*TraesCS7D02G106300*
